# GGTLC1 Is a Prognostic Biomarker in Renal Clear Cell Carcinoma

**DOI:** 10.1155/ijog/7524935

**Published:** 2025-08-12

**Authors:** Shi Li, Lijun Shi, Xiaofeng Tao, Guanghai Yu, Jianbo Wang

**Affiliations:** ^1^Department of Urology, First Affiliated Hospital of Dalian Medical University, Dalian, China; ^2^Department of Urology, Central Hospital of Dalian University of Technology, Dalian, China; ^3^Department of Minimally Invasive Surgical Ward of Cholecystitis Affiliated Zhongshan Hospital of Dalian University, Dalian, China; ^4^Department of Pathology, Central Hospital of Dalian University of Technology, Dalian, China

**Keywords:** GGTLC1, kidney renal clear cell carcinoma, KIRC, prognosis

## Abstract

Kidney renal clear cell carcinoma (KIRC), the most common type of renal cell carcinoma, is an immunobiologically and metabolically distinct tumor. Its clinical management is closely related to the tumor microenvironment (TME). Identifying its potential markers is important for improving prognosis. Therefore, in this study, we successfully constructed a prognostic model of glutathione metabolism–related molecules and screened for GGTLC1 prognostic molecules, while immunohistochemical analysis of the online database HPA and hospital patient specimens showed that GGTLC1 was highly expressed in paracancerous tissues. Taken together, the results of this study suggest that GGTLC1 is a potential prognostic biomarker associated with KIRC.

**Trial Registration:** Chinese Clinical Trial Registry identifier: LSKY: 2022-112-01

## 1. Introduction

Kidney renal clear cell carcinoma (KIRC) is the most common type of renal cell carcinoma, accounting for 75%–85% of all renal cell carcinoma patients, and its pathogenesis is related to heredity, smoking, obesity, and hypertension [1]. Most KIRC patients clinically treated with chemotherapy, radiotherapy, or targeted therapy have more obvious side effects, and surgical resection is still the main treatment at present [2]. Patients with KIRC have higher tumor recurrence and metastasis rates compared to patients with other renal cancer subtypes, and their poor prognosis is highly correlated with them [3]. In addition, the tumor microenvironment (TME) is extremely complex, consisting of immune cells, various types of stromal cells, and tumor cells [4]. It has been reported that the efficacy of immunotherapy for KIRC and the patient outcome are closely related to the heterogeneity of the tumor, and its surrounding TME is closely related [5]. Successful treatment of KIRC is challenging due to the inherently heterogeneous nature of the TME in KIRC patients. Therefore, we aimed to identify a prognostic biomarker that may help to explain the heterogeneity of the KIRC tumor immune microenvironment (IME), and it is significant to explore potential biomarkers for guiding the treatment of KIRC.

Metabolism-related signaling pathways are usually involved in the regulation of cell survival, cell growth, cell metabolism, protein synthesis, autophagy, and homeostasis [6, 7]. The pathological relevance of abnormal metabolic signaling has been explained in many human diseases, especially in various human cancers [8]. In chromophobe renal cell carcinoma (ChRCC), gamma-glutamyl transferase 1 (GGT1) expression is strikingly suppressed (nearly 100-fold lower than normal kidney), leading to markedly elevated intracellular glutathione (GSH) (GSH/GSSG) levels. GGT1 overexpression inhibits ChRCC cell proliferation in vitro and in vivo, suppresses cysteine uptake, and reduces GSH/GSSG levels. Low GGT1 expression promotes nuclear accumulation of NRF2, upregulating GCLC and GPX4 to enhance GSH synthesis. ChRCC cells depend on cysteine import via xCT (SLC7A11) to maintain high GSH, making them sensitive to cysteine uptake inhibitors. GGT1-mediated GSH metabolism creates a metabolic vulnerability, linking GGT1 deficiency to ferroptosis hypersensitivity in ChRCC, which may serve as a therapeutic target [9].

In this study, we focused on the effect of key prognostic molecules in the GSH metabolic pathway on KIRC, and we found that GGTLC1 was lowly expressed in KIRC by raw letter analysis, and the role of GGTLC1 in KIRC has still not received further attention. Therefore, our study initially focused on the relationship between GGTLC1 expression and clinicopathological features in patients with renal clear cell carcinoma, but the study of the molecular mechanism of GGTLC1 in renal clear cell carcinoma cells still needs further in-depth analysis.

## 2. Materials and Methods

### 2.1. Date Source and Processing

The 22 genes related to GSH metabolism investigated in this study were obtained from the Gene Set Enrichment Analysis (GSEA) package on the WikiPathways website (https://www.gseamsigdb.org/gsea/index.jsp). Additionally, RNA-Seq transcriptome data and associated clinical data on the patients with KIRC were obtained from TCGA, which contains 539 tumor samples and 72 normal samples. Over the past 10 years, 47 patients with KIRC who underwent surgical treatment in the Urology Department of Dalian Central Hospital were selected.

### 2.2. Least Absolute Shrinkage and Selection Operator (LASSO)–Cox Regression Analyses

We used the “correlogram” package to show coexpression relationships between any two GSH-related genes. Consistent with the approach of Xiangyu et al. [10], the “glmnet” package in RStudio was used to perform LASSO–Cox regression analyses to further identify the most useful prognostic genes and to establish a risk model. Next, we computed the risk score (RS) of each sample on the basis of the gene expression and coefficient values.

The RS for each patient was calculated as the sum of the products of normalized expression levels and their corresponding regression coefficients for all selected genes: risk *s*core = Exp_1_ × Coef_1_ + Exp_2_ × Coef_2_ + ⋯+Expi × Coefi. In this formula, Exp_i_ denotes the normalized expression level of gene i, Coef_i_ represents the LASSO-derived regression coefficient of gene i, and *n* is the total number of genes included in the final prognostic signature. Variable selection was performed via LASSO regression, with the optimal model identified at lambda.min (*λ* = 0.023) through 10-fold cross-validation. We used the “survminer” package to obtain the optimal thresholds for dividing the sample into high-risk and low-risk groups and used the “survival” package in R to calculate the survival curves for these two groups. The survival ROC package in R was used to generate the subject operating characteristic (ROC) curves and to obtain the area under the ROC curves.

### 2.3. Construction of a Nomogram for Predicting the Outcome of Patients KIRC

Univariate and multivariate Cox regression analyses were used to determine RSs in models for patient age, tumor stage, and tumor grading. Finally, the results of the Cox analyses were plotted against the clinical data in a nomogram evaluating the characteristic KIRC for patient survival probability.

### 2.4. The Human Protein Atlas

HPA (https://www.proteinatlas.org/) is an open access protein database. An immunohistochemical method was used to detect the expression level of GGTLC1 protein in renal clear cell carcinoma and normal tissues.

### 2.5. Immunohistochemistry (IHC)

Paraffin-embedded specimens were used to assess GGTLC1 expression in the paracarcinomas of 47 KIRC patients and nine tumor patients. Briefly, after xylene immersion and dehydration with different purities of alcohol, antigen repair was performed using hydrogen peroxide (0.3%) to block cellular peroxidase activity and normal goat serum at 37°C to block the site of nonspecific immunoglobulin interactions. The primary antibody was incubated overnight at 4°C, followed by the secondary antibody at room temperature and finally DAB for color development.

### 2.6. BEST Online Analysis

Sequencing data and clinical prognostic data from multiple cohorts of KIRC were summarized by the BEST tool (https://rookieutopia.com/). Subsequently, the gene was analyzed for high and low expression pathway enrichment, and the prognosis of GGTLC1 in KIRC was focused.

### 2.7. Association of GGTLC1 With Immune Cell Tumor Infiltration and Drug Sensitivity

ESTIMATE scores were used to calculate the stromal score, immune score, and estimated score to assess the presence of stromal cells and infiltration of immune cells. Therefore, we compared the immune infiltration of GGTLC1 with high and low expression in KIRC patients in TCGA. Then, data related to KIRC immunotherapy scores were downloaded from the TCIA (https://tcia.at/) database to compare immunotherapy in the GGTLC1 high and low expression groups. Finally, drug sensitivity analysis of GGTLC1 high and low expression groups was done based on the Genomics of Drug Sensitivity in Cancer (GDSC) database.

### 2.8. Statistical Analysis

All statistical analyses were performed using RStudio. Survival curves were analyzed using the Kaplan–Meier method. The two groups were analyzed using a two-tailed *t*-test. *p* values < 0.05 were considered statistically significant differences.

## 3. Result

### 3.1. Risk Model Construction Using LASSO–Cox Regression Analyses

**Figure 1:**
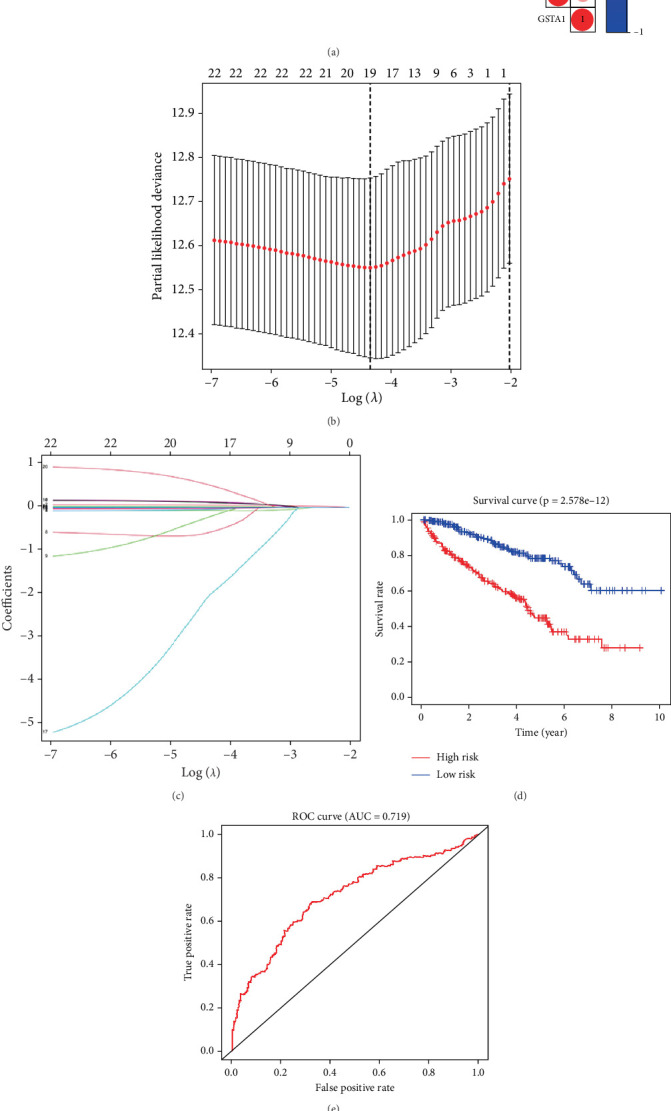
Risk models were constructed using the least absolute shrinkage and selection operator (LASSO)–Cox regression analysis. (a) Graph showing the results of coexpression relationships of 22 glutathione metabolism–related genes in KIRC. In the color bar on the right, red represents positive correlation and blue represents negative correlation. (b) LASSO coefficient profiles of glutathione pathway genes in KIRC. (c) Distribution and median of risk scores. (d) Survival curves obtained from the model. The overall survival rate of patients in the high-risk group was significantly lower than that of patients in the low-risk group. (e) ROC curve at 5-year year with an area under the curve (AUC) of 0.719. (f) Correlation between risk scores and clinicopathological characteristics (*p* < 0.001).

To explore the relationship between genes in the GSH metabolism–related pathway, we performed coexpression analysis of 22 GSH metabolism–related genes, and the results are shown in (Figure 1a). To determine whether GSH metabolism–related genes could be used to build a clinical prognostic prediction model for KIRC patients, LASSO–Cox regression analyses was performed on the 22 genes from their coexpression analysis, and 19 genes were finally selected to construct a RS model (Figure 1b,c). Patients with KIRC were divided into high-risk and low-risk groups according to their RS values. The overall survival rate of patients in the high-risk group was significantly lower than that of patients in the low-risk group (*p* < 0.001, Figure 1d). To explore the prognostic predictive efficiency of the new survival model for patients with KIRC, we performed a ROC curve analysis. The area under the ROC curve for the survival model to predict 5-year survival was 0.719, indicating the high predictive value of the risk model (Figure 1e). Further statistical tests were performed on the differences between risk subgroups, and heat maps were drawn to visualize the correlation between RS and clinical data (Figure 1f). We found that the risk model correlated with tumor metastasis (M), tumor size (T), stage, and grade, with patients in the high-risk group tending to have advanced histological grade and advanced clinical stage, while those in the low-risk group tended to have the opposite.

### 3.2. Evaluating the Prognosis of KIRC Patients by Nomogram

**Figure 2:**
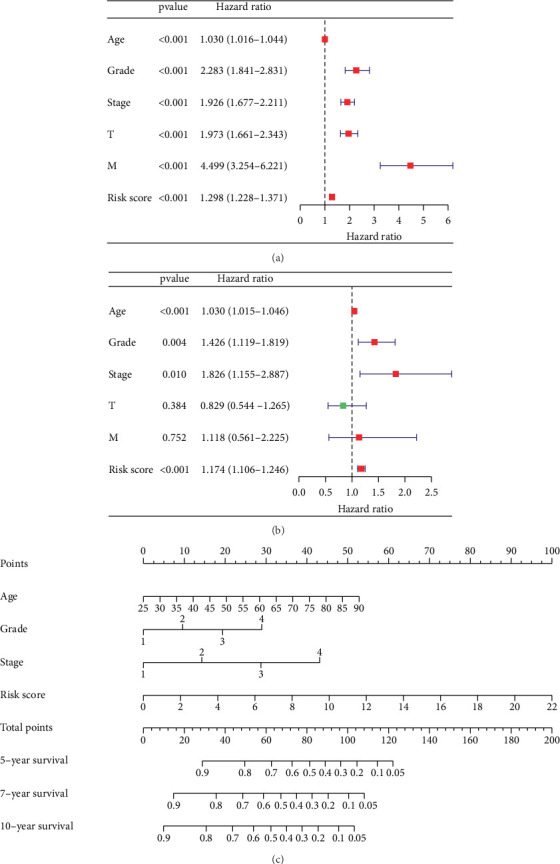
A nomogram was used to assess the prognosis of KIRC patients. (a) Analysis using univariate Cox regression of risk scores (RSs) showed that age, tumor grade, stage, T, M, and RS were correlated with patients' OS (*p* < 0.05). (b) Analysis using multifactorial Cox regression of risk scores (RSs) showed that age, tumor grade, stage, and RS were associated with patients' OS (*p* < 0.05). (c) The nomogram contains risk score, age, grade, and stage, which can be used to predict the prognosis of KIRC patients.

We performed univariate Cox regression analysis and multivariate Cox regression analysis on RS and other clinicopathological characteristics of KIRC patients (Figure 2a,b). Among them, patient age, tumor grade, and RS were independent risk factors associated with overall survival. The nomogram drawn by the “rms” package in RStudio incorporated RS, age, grade, and stage, which can be used to predict the outcome of patients with KIRC (Figure 2c). For example, if the overall score is 100, the 5-year survival rate is approximately 0.25.

### 3.3. GGTLC1 Is Lowly Expressed in KIRC and Correlates With Clinical Characteristics and Prognosis of Patients

**Figure 3:**
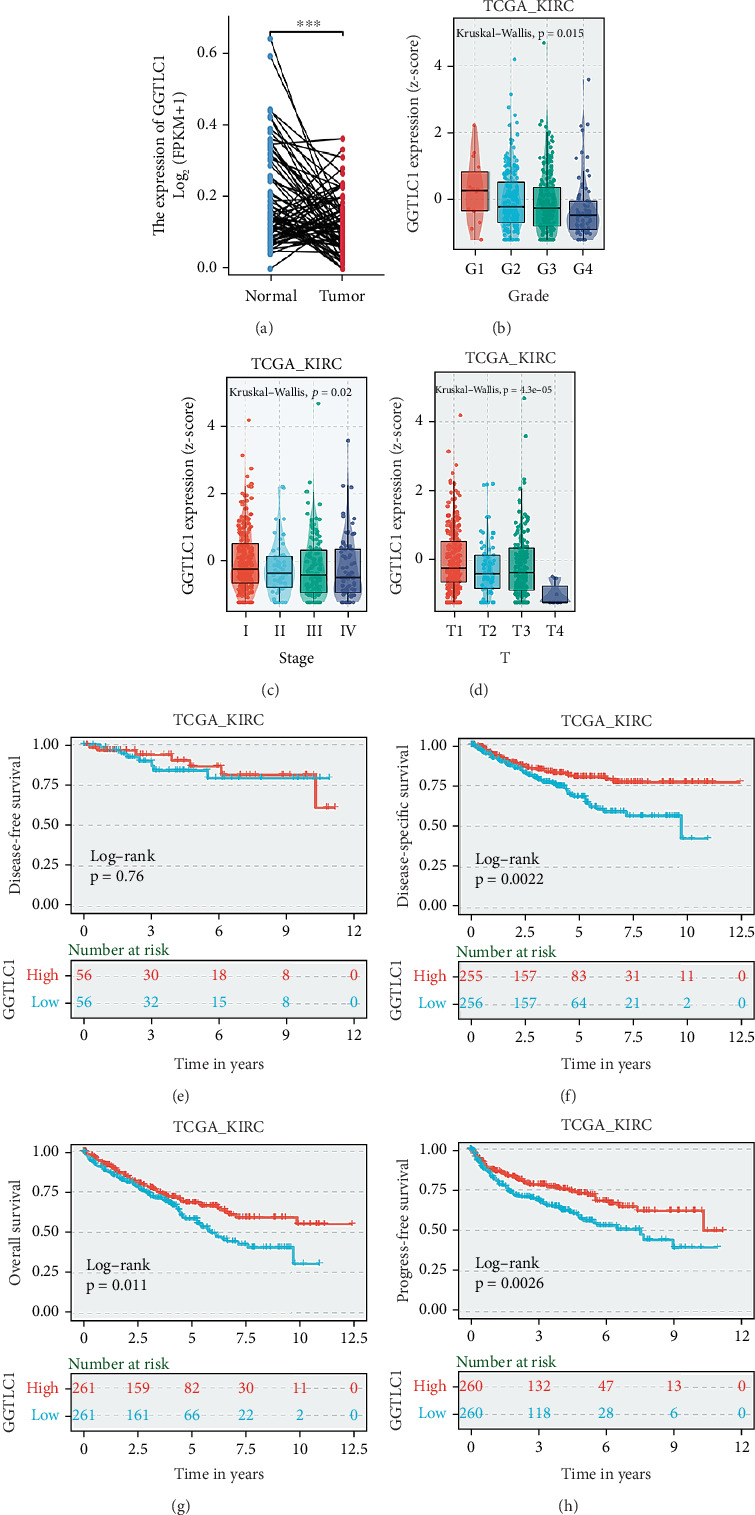
GGTLC1 is lowly expressed in KIRC and correlates with its clinicopathological features and prognosis. (a) Expression levels of GGTLC1 in normal and tumor tissues paired in the TCGA database. (b–d) Expression of GGTLC1 in KIRC in different grades (G1, G2, G3, andG4), stages (I, II, III, and IV), and T-stages (T1, T2, T3, and T4). (e–h) GGTLC1 high expression in patients with clear cell carcinoma of the kidney demonstrating better disease-specific survival (DSS), overall survival (OS), and progression-free survival (PFS).

**Figure 4:**
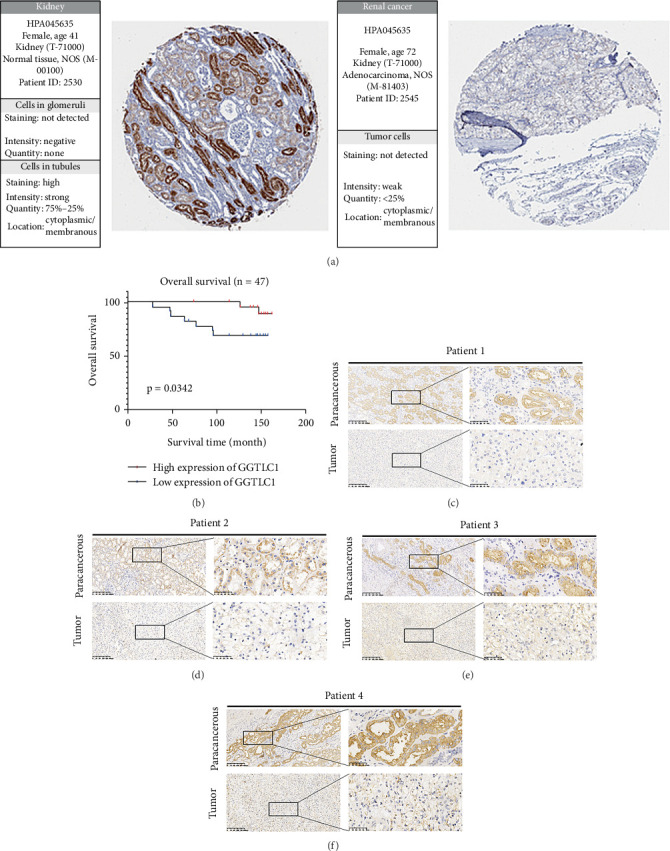
GGTLC1 has low protein expression in KIRC. (a) Protein expression levels of GGTLC1 in paired normal and tumor tissues from the HPA database. (b) The Kaplan–Meier curve for the prognosis of KIRC. (c–f) Protein expression levels of GGTLC1 in normal and tumor tissues of four patients with KIRC from Dalian Municipal Central Hospital.

First, we examined the expression levels of GGTLC1 in normal and tumor tissues paired with the TCGA database and found that GGTLC1 was highly expressed in normal samples (Figure 3a). We then analyzed the expression of GGTLC1 in different grades (G1, G2, G3, and G4), stages (I, II, III, and IV) and T-stages (T1, T2, T3, and T4) in KIRC using the online website BEST database (Figures 3b, 3c, and 3d). Our data showed that the expression of GGTLC1 was reduced in G4, IV, and T4 stages. In addition, KIRC patients with low GGTLC1 expression had a poorer prognosis, including OS, disease-specific survival (DSS) and progression-free interval (PFI) (Figures 3e, 3f, 3g, and 3h). In addition, patients were divided into high and low expression groups based on the order of scoring of each paraffin section. Kaplan–Meier curves showed that high expression of GGTLC1 was associated with a favorable prognosis (*p* = 0.0342, Figure 4b). Finally, we compared the protein expression levels of GGTLC1 in normal and tumor tissues. Our results revealed low levels of GGTLC1 protein expression in KIRC patients in the HPA public database (Figure 4a). Similarly, we examined cancer and paracancer samples from four KIRC patients in Dalian Central Hospital, which all showed low protein expression levels of GGTLC1 in tumors (Figures 4c, 4d, 4e, and 4f).

### 3.4. Metabolism-Related Pathways Regulated by GGTLC1 in KIRC

**Figure 5:**
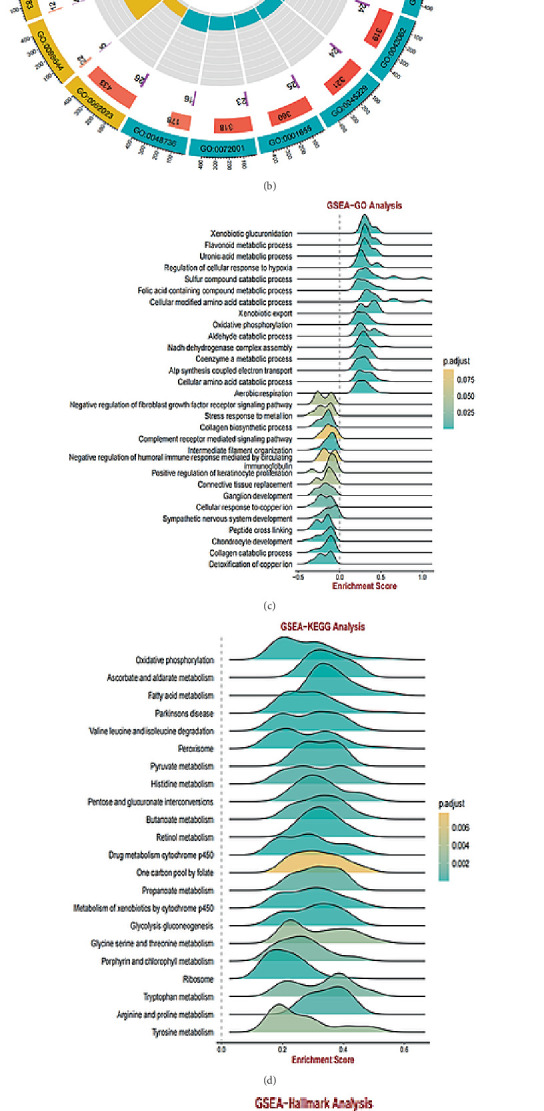
GO and KEGG enrichment analyses of coexpressed genes of GGTLC1. (a) GGTLC1 coexpressed significantly related 10 genes. (b) Functional annotation of GGTLC1 from biological processes, cellular components, and molecular functions by GO enrichment analysis. (c–e) GO, KEGG, and hallmark enrichment analyses of GGTLC1 were analyzed by the BEST online website, and the results revealed that they were mainly related to metabolism.

To explore the biological functions and roles played by GGTLC1 in KIRC, we performed KEGG and GO analyses. First, we identified differential genes associated with high and low expression of GGTLC1 in KIRC, in which 10 significantly associated genes were listed (Figure 5a). Then, GO pathway enrichment was performed to explore the biological processes associated with high GGTLC1 expression in KIRC (Figure 5b). We then further explored the major pathways through which GGTLC1 exerts its function using the BEST public database (Figures 5c, 5d, and 5e). Our results showed that GGTLC1 was mainly associated with the activation of metabolism-related pathways, such as oxidative phosphorylation, adipogenesis, fatty acid metabolism, and bile acid metabolism.

### 3.5. Relationship Between Drug and Immunotherapy Sensitivity and High and Low GGTLC1 Expression

**Figure 6:**
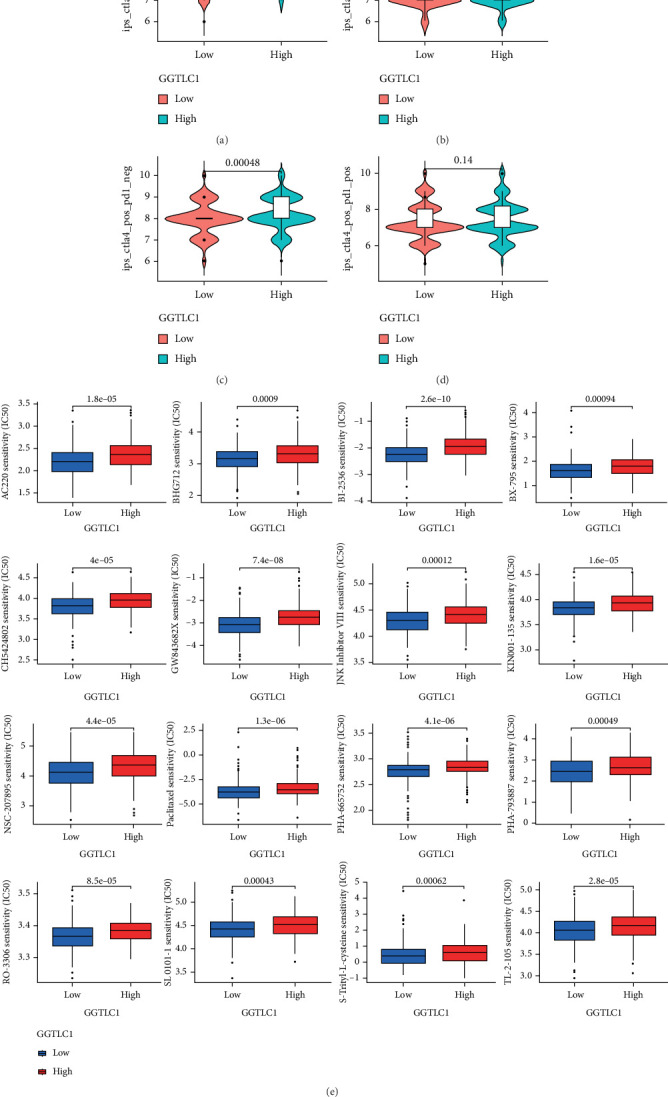
Relationship between drug and immunotherapy sensitivity and high and low GGTLC1 expression. (a–d) Correlation between immunotherapy effect and GGTLC1 expression. (e) Differences between drug sensitivity and high and low GGTLC1 expression for 16 drugs.

Molecularly targeted therapies and immunotherapy are currently common treatments for KIRC. We downloaded data related to KIRC immunotherapy scores from the TCIA (https://tcia.at/) database (Figures 6a, 6b, 6c, and 6d) as well as assessed the degree of drug sensitivity of GGTLC1 in high and low expression of KIRC using the R package (pRRophetic) (Figure 6e). Our results showed that anti-CTLA4 treatment was more effective when GGTLC1 was highly expressed; meanwhile, low GGTLC1 expression might be sensitive to 16 drugs such as BHG712 (an inhibitor of EphB4 kinase autophosphorylation) and BI-2536 (an inhibitor of INF-*β*).

## 4. Discussion

GSH, a tripeptide thiol antioxidant composed of glutamate, cysteine, and glycine [11], is a major ROS scavenger in cells. Also, GSH has several other functions including, but not limited to, involvement in iron–sulfur protein maturation [12], modulation of protein bioactivity through S-glutathionylation [13], and regulation of redox signaling [14]. In recent years, it has been found that GSH metabolism–related metabolites (including cysteine, *γ*-glutamylcysteine, and GSH) are shown to be increased in advanced KIRC and correlate with poorer survival outcomes in patients with KIRC [15–17]. ChRCC shows hypersensitivity to ferroptosis due to GSH metabolic dependency and cysteine import via SLC7A11. Low GGT1 expression maintains high GSH levels, while GGT1 overexpression inhibits ChRCC growth. Inhibiting cysteine uptake or using cystinase induces ferroptosis, offering a targeted therapy approach.

Based on the results of existing studies, we firstly constructed a prognostic model of KIRC using GSH-related genes and explored the predictive effect of the new survival model on the prognosis of KIRC patients. The results of subsequent bioinformatics analyses indicated that GGTLC1 might be a new KIRC prognostic biomarker. Previous studies have reported that GGT1 is involved in oxidative stress, ROS production, and tumor cell proliferation dense [18]. In addition, GGTLC1 is a member of the glutamyltransferase family and encodes the light chain portion of GGT, the catalytically active part of the GGT1 protein [19]. A study finds GGTLC1 upregulated in endometrial cancer, inhibiting its progression by regulating the TGF-*β*/Smad pathway, a potential target for treatment [20]. GGT1 is a transmembrane protein that plays an important role in cellular functions such as proliferation, apoptosis, migration, and immune homeostasis [21, 22]. GGT1 is a ligand for signal regulatory protein alpha (SIRP*α*), which is present on immune cells such as macrophages and dendritic cells. SIRP*α* binds to GGT1 to initiate a signaling cascade and ultimately inhibit phagocytosis [23]. Blocking the GGT1 signaling pathway activates phagocytosis and promotes tumor eradication. However, the relationship between GGT1, GGTLC1, and the IME of KIRC and the mechanisms leading to tumor heterogeneity have not been fully investigated.

The IME is critical to the etiology of renal clear cell carcinoma and patient prognosis [24]. ccRCC has an immune infiltrate composed mainly of T cells, with a high ratio of CD8+ T cells to regulatory T cells (Tregs). However, ccRCC also has a high density of MDSCs, which can suppress the antitumor immune response [25, 26]. In addition, ccRCC tumors have increased expression of immune checkpoint molecules, such as programmed death ligand 1 (PD-L1), which inhibits T cell activation and promotes tumor immune evasion [5, 27]. In addition, due to the glycolipid metabolism of renal cell carcinoma, this cancer may be accompanied by various types of resistance to tyrosine kinase inhibitors (TKIs) and immune checkpoint inhibitors (ICIs). With the increase of lactate, nitric oxide, and other new metabolic byproducts, the new discovery of TMEs and key metabolic enzymes driven by HIF and other factors is becoming increasingly clear in the oncogenesis and treatment of renal cell carcinoma. Therefore, the relationship between metabolism-related molecules and the IME deserves in-depth study.

Based on our current findings, GGTLC1 may act as an antioncogene metabolic molecule. It has not been reported to have a specific mechanism associated with tumors within the past years, so the subsequent antioncogenic function in KIRC still needs further investigation. Meanwhile, it is also important for us to elucidate how GGTLC1 affects the progression of KIRC through immune infiltration and TME. Currently, there are significant unanswered questions in evaluating biomarker-based predictions of immunotherapy efficacy. Therefore, these may be the focus of future studies, which will provide a basis for the use of GGTLC1 as a prognostic biomarker for KIRC.

## Data Availability

The datasets analyzed during the current study are available in the TCGA. The link is as follows: https://portal.gdc.cancer.gov/projects/TCGA-KIRC. The data used to support the findings of the study can be obtained from the corresponding author upon request.
